# Eph-B4 regulates adaptive venous remodeling to improve arteriovenous fistula patency

**DOI:** 10.1038/s41598-017-13071-2

**Published:** 2017-11-13

**Authors:** Clinton D. Protack, Trenton R. Foster, Takuya Hashimoto, Kota Yamamoto, Monica Y. Lee, Jan R. Kraehling, Hualong Bai, Haidi Hu, Toshihiko Isaji, Jeans M. Santana, Mo Wang, William C. Sessa, Alan Dardik

**Affiliations:** 1 Vascular Biology and Therapeutics Program, Yale School of Medicine, New Haven, CT USA; 2 Department of Surgery, Yale School of Medicine, New Haven, CT USA; 3 Department of Surgery, VA Connecticut Healthcare System, West Haven, CT USA; 4 Department of Vascular Surgery, The University of Tokyo, Tokyo, Japan; 5 Department of Pharmacology, Yale School of Medicine, New Haven, CT USA

## Abstract

Low rates of arteriovenous fistula (AVF) maturation prevent optimal fistula use for hemodialysis; however, the mechanism of venous remodeling in the fistula environment is not well understood. We hypothesized that the embryonic venous determinant Eph-B4 mediates AVF maturation. In human AVF and a mouse aortocaval fistula model, Eph-B4 protein expression increased in the fistula vein; expression of the arterial determinant Ephrin-B2 also increased. Stimulation of Eph-B-mediated signaling with Ephrin-B2/Fc showed improved fistula patency with less wall thickness. Mutagenesis studies showed that tyrosine-774 is critical for Eph-B4 signaling and administration of inactive Eph-B4-Y774F increased fistula wall thickness. Akt1 expression also increased in AVF; Akt1 knockout mice showed reduced fistula diameter and wall thickness. In Akt1 knockout mice, stimulation of Eph-B signaling with Ephrin-B2/Fc showed no effect on remodeling. These results show that AVF maturation is associated with acquisition of dual arteriovenous identity; increased Eph-B activity improves AVF patency. Inhibition of Akt1 function abolishes Eph-B-mediated venous remodeling suggesting that Eph-B4 regulates AVF venous adaptation through an Akt1-mediated mechanism.

## Introduction

Understanding venous remodeling remains a significant clinical challenge. For example, arteriovenous fistulae (AVF) remain the gold standard for hemodialysis access in patients with end stage renal disease yet outcomes for AVF are poor, with up to 60% of fistulae failing to attain suitability for dialysis^1^. Failure of fistula maturation requires patients to have repeated interventions and increases catheter-based dialysis^2,3^. A greater understanding of the molecular events guiding successful venous remodeling is needed to develop strategies to improve AVF maturation^4^.

Studies of vein graft remodeling provide some understanding of venous adaptation to the arterial environment. Vein graft remodeling is stimulated by mechanisms that are thought to be similar to those active after blood vessel injury^5–7^. Human studies using high resolution imaging have documented venous wall thickening and outward remodeling as consistent components of successful venous remodeling^8^. Venous remodeling is characterized by increased deposition of smooth muscle cells and changes in extracellular matrix component expression resulting in wall thickening and reduced compliance^9,10^. However, it is not currently understood which aspects of remodeling are critical for successful venous adaptation to the arterial environment, whereas excessive remodeling may result in neointimal hyperplasia and clinical failure. The disappointing results of the PREVENT-III and -IV trials suggest that strategies to prevent vein graft failure that focus on inhibition of smooth muscle cell proliferation are not likely to be clinically useful^11,12^.

Erythropoietin-producing hepatocellular carcinoma (Eph) receptors are the largest family of tyrosine kinase receptors with an essential role during embryonic vascular development^13^. Eph receptors and their Ephrin ligands exhibit differential expression patterns determining venous and arterial identity^14^; venous endothelial cells are characterized by Eph-B4 expression, while arterial endothelial cells preferentially express Ephrin-B2^15,16^. Eph receptor activation triggers phosphorylation and downstream signaling; in particular, Eph-B4 activates the PI3K-Akt pathway to promote cell migration and proliferation *in vitro* ^17^. Akt is a regulator of cellular metabolism affecting protein synthesis, cell growth, and promoting cell survival, all of which are essential functions that are critical for venous remodeling^18^. We have previously shown that Akt expression, but not phosphorylation, is upregulated during vein graft adaptation^19,20^, suggesting a potential role for Akt during venous remodeling.

During successful vein graft adaptation we previously reported that Eph-B4 expression decreases in human vein grafts as well as in a rat model, e.g. venous remodeling in the arterial environment is characterized by loss of venous identity without gain of arterial identity^7^. Eph-B4 expression similarly decreased in a mouse vein graft model; loss of Eph-B4 function resulted in venous wall thickening, whereas activation of Eph-B4 with Ephrin-B2/Fc, a specific bivalent ligand, resulted in inhibited remodeling with reduced venous wall thickening^20^. These findings suggest that Eph-B4 promotes vein graft adaptation by negatively regulating venous wall thickening. However, it is not clear whether Eph-B4 specifically mediates vein graft adaptation or whether it mediates adaptive venous remodeling in general; it is also not clear whether loss of vessel identity is a general characteristic of venous remodeling or specific to vein graft adaptation.

To directly test whether Eph-B4 regulates adaptive venous remodeling, and how vessel identity changes during venous remodeling, we examined AVF maturation. Successful AVF maturation also requires venous adaptation to a fistula environment that is similar to the arterial environment but is characterized by different hemodynamic forces, disturbed flow, and different venous wall oxygen tension compared to vein graft adaptation^10^. We hypothesized that Eph-B4 mediates AVF maturation; we also hypothesized that Akt is a critical regulator of Eph-B4-mediated venous remodeling.

## Results

### Gain of dual arterial-venous identity during adaptive venous remodeling

**Figure 1:**
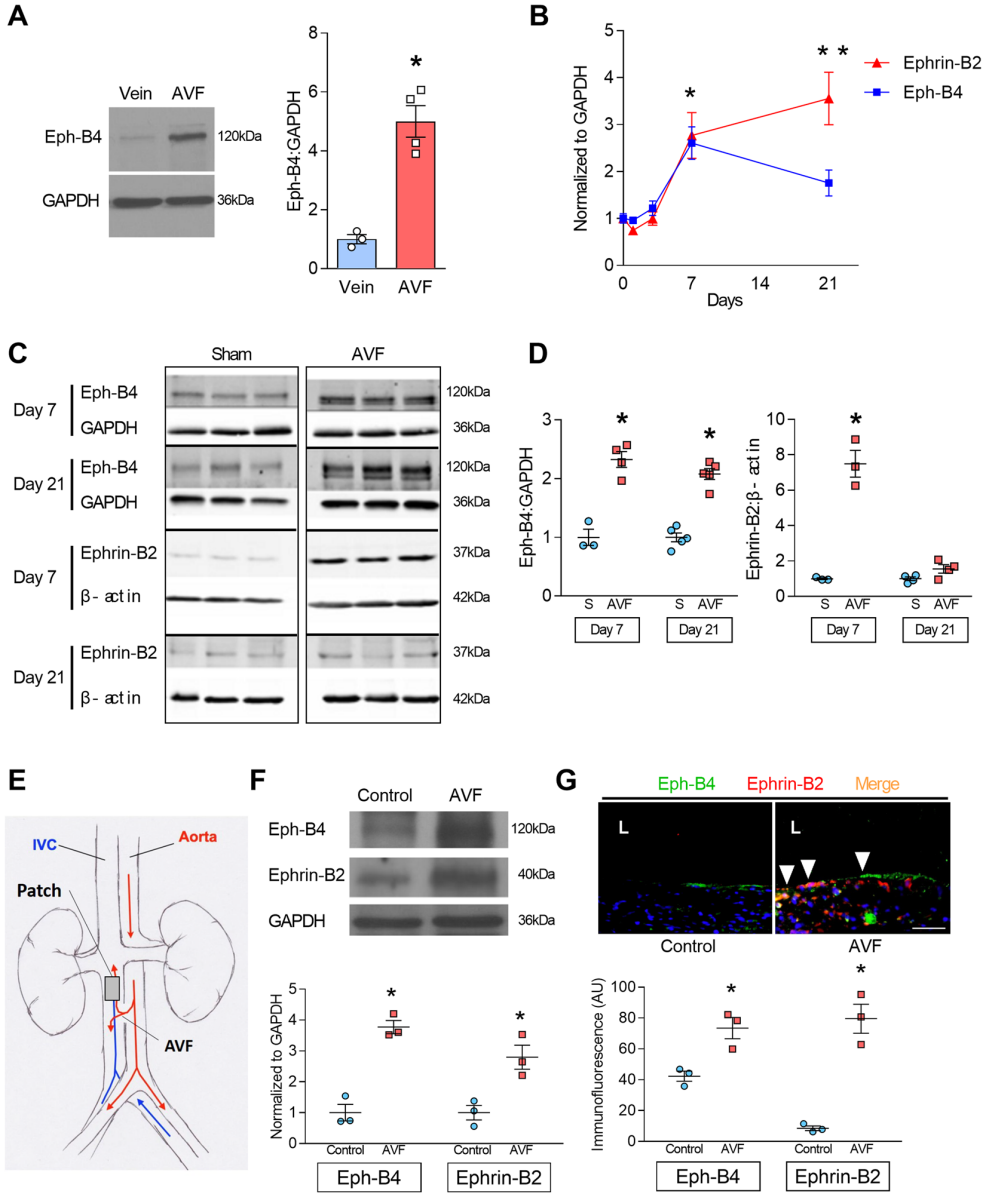
Increased Eph-B4 and Ephrin-B2 expression during adaptive venous remodeling. (**A**) Western blot and adjacent bar graph of densitometry showing human Eph-B4 expression in AVF venous limb compared to normal vein. *P = 0.0016; t-test. *n* = 3–4. (**B**) Line graphs show expression of *Eph-B4* (blue) and *Ephrin-B2* (red) in the AVF venous limb compared to sham IVC; *P* < 0.0001 (ANOVA). *P < 0.05 (*P* = 0.0123, *Eph-B4*; *P* = 0.0041, *Ephrin-B2*; post hoc); **P < 0.05 (*P* < 0.0001, *Ephrin-B2*; post hoc). *n* = 5–8. (**C**) Western blots showing Eph-B4 and Ephrin-B2 protein expression in AVF venous limb compared to sham IVC. *n* = 3–5. (**D**) Graphs showing densitometry of Eph-B4 (left panel) and Ephrin-B2 (right panel) expression in the AVF venous limb compared to sham IVC; *P < 0.05 (*P* < 0.0001, Eph-B4 day 7, AVF vs sham; *P* < 0.0001, Eph-B4 day 21, AVF vs sham; *P* < 0.0001, Ephrin-B2 day 7, AVF vs sham; post hoc). *n* = 3–5. (**E**) Diagram of rat model showing location of infrarenal IVC pericardial patch exposed to an aortocaval AVF (n = 6 per group). (**F**) Representative Western blot (upper panel) showing Eph-B4 and Ephrin-B2 expression in patch neointima (day 14) of control vein compared to patch neointima of AVF vein. Graphs (lower panel) show quantification of western blot bands; *P* < 0.0001 (ANOVA). *P < 0.05 (*P* = 0.0003, Eph-B4; *P* = 0.0043, Ephrin-B2; post hoc). *n* = 3. (**G**) Representative photomicrographs (upper panel) showing Eph-B4 (green) and Ephrin-B2 (red) immunoreactive signal (day 14). White arrowheads indicate colocalization of Eph-B4 and Ephrin-B2. L, vessel lumen. Graph (lower panel) shows quantification of immunoreactive signal; *P* < 0.0001 (ANOVA). *P < 0.05 (*P* = 0.0136 Eph-B4; *P* < 0.0001 Ephrin-B2; post hoc). *n* = 3. Scale bar 100 µm. Data represent mean ± SEM.

We previously showed that patent human vein grafts have reduced Eph-B4 expression, e.g. they lose venous identity without gaining arterial identity^7^. However, successfully matured human AVF showed increased Eph-B4 expression relative to normal veins from the same patients (Fig. 1A). To determine the time course of changes in Eph-B4 expression during venous adaptation, Eph-B4 expression was determined after surgical creation of an infrarenal aortocaval fistula in WT mice. During AVF maturation, mRNA transcription of the venous determinant *Eph-B4* increased compared to basal levels, with maximal expression at day 7 (Fig. 1B, blue line). Similarly, there was increased mRNA transcription of the arterial determinant *Ephrin-B2*, with maximal expression at day 21 (Fig. 1B, red line). Expression of several other venous and arterial markers of identity was similarly increased (Table 1). Western blot of the venous limb of the AVF showed increased Eph-B4 and Ephrin-B2 protein expression at day 7, with sustained increased Eph-B4 expression at day 21 (Fig. 1C and D). These results are consistent with acquisition of dual arterial-venous identity during AVF maturation in the fistula environment, which is distinctly different than the loss of vessel identity during vein graft adaptation to the arterial environment.

**Table 1:** Expression of vessel identity markers in mouse AVF compared to sham veins, days 7 and 21 (n = 8).

Marker	p-value^*^	7 days	p-value^**^	21 days	p-value^**^
**Venous**					
Eph-B4	<0.0001	2.605 ± 0.3456	0.0002	1.754 ± 0.2766	0.124
Neuropilin2	0.009	1.365 ± 0.262	0.9225	2.71 ± 0.4512	0.0073
**Arterial**					
Ephrin-B2	<0.0001	2.768 ± 0.489	0.0150	3.557 ± 0.558	0.0003
VEGF-R2	<0.0001	1.388 ± 0.2069	0.4941	2.325 ± 0.2745	0.0001
Jagged1	0.0001	2.625 ± 0.4426	0.0079	2.622 ± 0.457	0.0080
Dll4	0.0005	1.29 ± 0.2524	0.8682	2.054 ± 0.4052	0.0187
Hey2	<0.0001	2.786 ± 0.3775	0.0034	2.726 ± 0.5887	0.0047
Neuropilin1	<0.0001	1.388 ± 0.2069	0.4941	2.325 ± 0.2745	0.0001
Notch-1	<0.0001	2.062 ± 0.2682	0.0143	2.002 ± 0.3841	0.0225
CD44	0.0001	4.419 ± 0.5585	0.0051	3.325 ± 0.5885	0.0849
Unc5b	0.0015	6.298 ± 1.23	0.0034	4.102 ± 1.635	0.1417

^*^, 1-way ANOVA; **, Dunnett’s post-hoc test.

Since both Eph-B4 and Ephrin-B2 expression are increased during AVF maturation but not during vein graft adaptation^7^, we determined the expression of Eph-B4 and Ephrin-B2 in another model that exposes neointimal formation to AVF flow^21^. A bovine pericardial patch was surgically placed into a rat infrarenal IVC without and with AVF creation (Fig. 1E). Neointima that formed on the luminal surface of the patches showed increased Eph-B4 and Ephrin-B2 protein expression (day 14) with the addition of a fistula (Fig. 1F **)**. Increased Eph-B4 and Ephrin-B2 immunoreactivity with the addition of a fistula was also detected by immunofluorescence (Fig. 1G), and these signals colocalized (white arrowheads). In both the mouse aortocaval and rat patch models there is increased phosphorylated tyrosine (pTyr) immunoreactivity in the presence of an AVF but this signal did not colocalize with the increased Eph-B4 immunoreactivity (Supplementary Figure 1, white arrows show unmerged Eph-B4 reactivity). These data show that in two different models, the presence of an AVF increases both Eph-B4 and Ephrin-B2 expression in the vein, e.g. venous remodeling in the fistula environment is characterized by dual arterial-venous identity.

### Activation of Eph-B4 regulates adaptive venous remodeling

**Figure 2:**
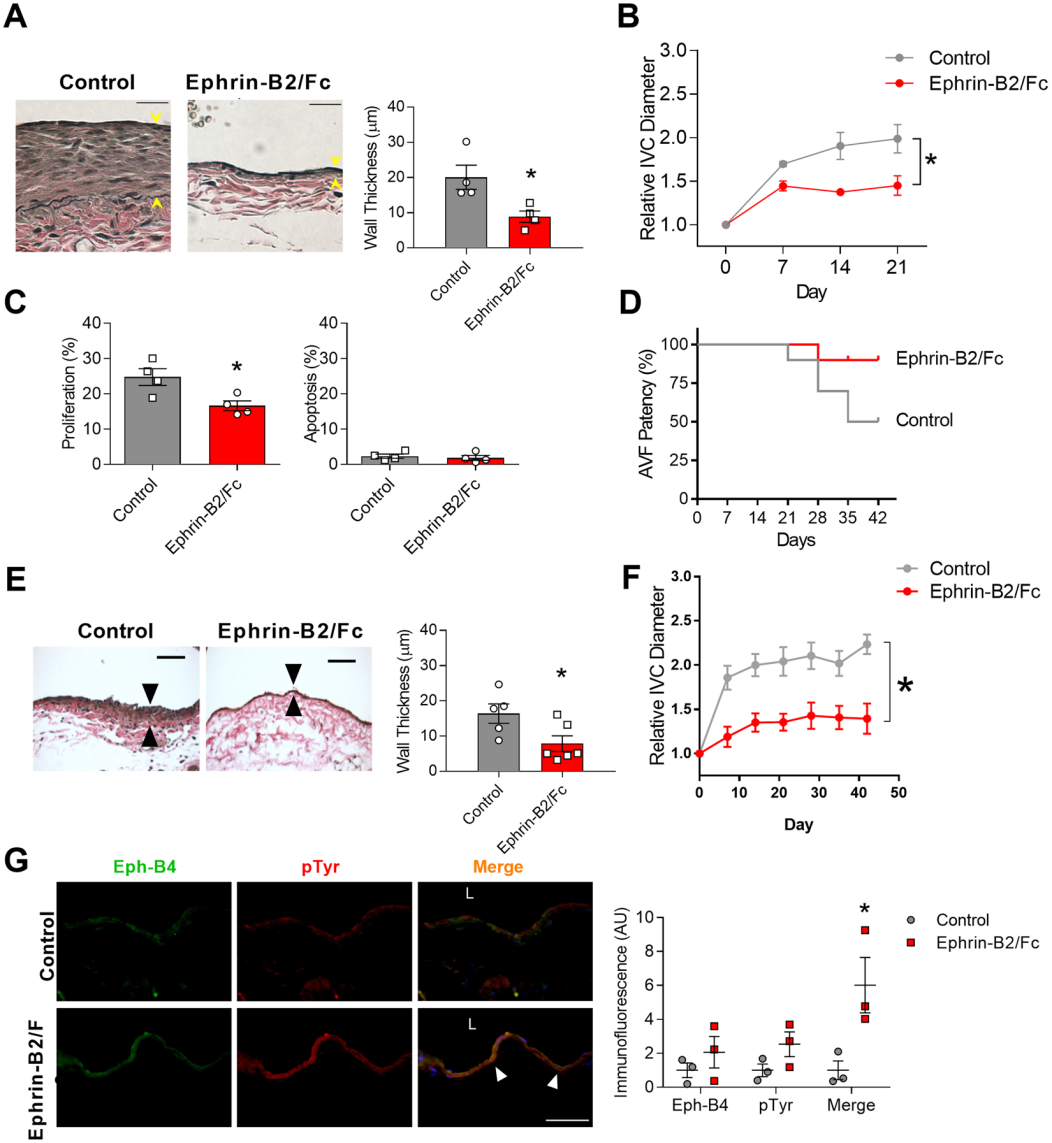
Activation of Eph-B4 regulates venous remodeling. (**A**) Representative photomicrographs (left panel) and bar graph (right panel) showing AVF venous wall thickness in AVF treated with control or Ephrin-B2/Fc (day 21); *P = 0.03 (t-test). *n* = 4. Scale bar, 25 µm. (**B**) Line graph showing IVC diameter in mice treated with control or Ephrin-B2/Fc; *P < 0.0001 (ANOVA). *n* = 4–5. (**C**) Bar graphs showing percentage of cells in the AVF positive for proliferation (Ki67) or apoptosis (cleaved caspase-3) (day 21); *P = 0.02 (t-test). *n* = 4. (**D**) Line graph showing AVF patency in mice treated with control or Ephrin-B2/Fc injections. *P* = 0.05 (Chi-square); *P* = 0.06 (Log-rank), *n* = 10. (**E**) Representative photomicrographs (left panel) and bar graph (right panel) showing AVF venous wall thickness in AVF treated with control or Ephrin-B2/Fc (day 42); *P = 0.04 (t-test). *n* = 5–6. Scale bar, 50 µm. (**F**) Line graph showing IVC diameter in mice treated with control or Ephrin-B2/Fc; *P < 0.0001 (ANOVA). *n* = 13–14. (**G**) Representative photomicrographs (left panel) and graph (right panel) showing Eph-B4 (green), phosphotyrosine (pTyr; red), and merged (yellow) immunoreactive signal with control (top row) or Ephrin-B2/Fc (bottom row) (day 21). Yellow arrowheads indicate merged signal. *P* = 0.0042 (ANOVA). *P = 0.0052 (post hoc). *n* = 3. Scale bar, 100 µm. Data represent mean ± SEM.

We determined whether this increased Eph-B4 in the AVF would be capable of function by stimulating Eph-B4 *in vivo* with its specific ligand, Ephrin-B2/Fc^20^. Control AVF showed thickening of the venous wall (day 21) that was not seen in AVF treated with Ephrin-B2/Fc (Fig. 2A). Control AVF showed outward remodeling of the venous limb that was reduced in AVF treated with Ephrin-B2/Fc (Fig. 2B) although there was no difference in aortic limb remodeling (Supplementary Figure 2). AVF treated with Ephrin-B2/Fc showed reduced numbers of proliferating cells with no increase in apoptotic cells in the venous limb (Fig. 2C). In the mouse AVF model, between postoperative days 21 and 42 there is increased neointimal hyperplasia with wall thickening and reduced patency, similar to the neointimal hyperplasia leading to early failure of human AVF^22^; however AVF treated with Ephrin-B2/Fc demonstrated improved fistula patency by day 42 with little thickening of the fistula venous limb wall as well as little outward remodeling (Fig. 2D–F). Mice treated with Ephrin-B2/Fc also showed colocalization of Eph-B4 and pTyr immunoreactivity in the venous limb of the AVF (Fig. 2G). These results suggest that activated Eph-B receptors in the AVF regulate both wall thickening and outward remodeling during AVF maturation and that improved AVF patency with Ephrin-B2/Fc stimulation may be due to improved maturation and/or less neointimal hyperplasia.

### Reduced Eph-B4 activity increases venous neointimal thickening

**Figure 3:**
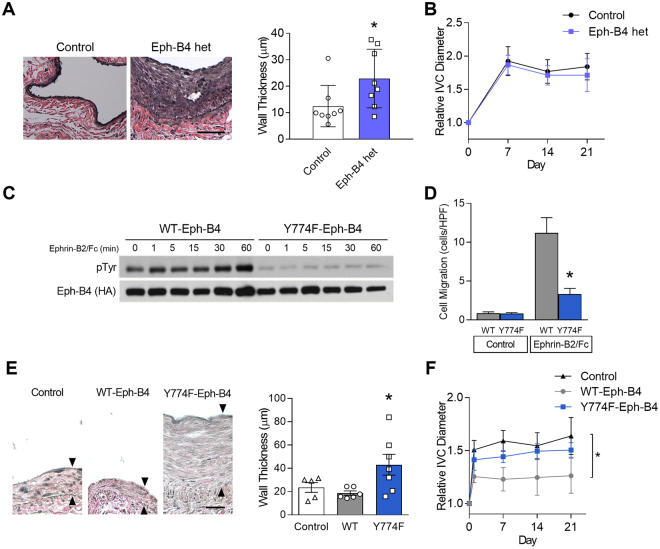
Reduced Eph-B4 activity increases venous neointimal thickening. (**A**) Representative photomicrographs (left panel) and bar graph (right panel) showing AVF venous limb wall thickness in control and Eph-B4 het mice (day 21); *P = 0.047 (t-test). *n* = 8. Scale bar 25 µm. (**B**) Line graph showing infrarenal IVC diameter in control or Eph-B4 het mice; *P = 0.59 (ANOVA). *n* = 8–9. (**C**) Representative Western blot showing inhibited tyrosine phosphorylation in the Y774F-Eph-B4 mutant compared to the WT-Eph-B4 construct (0–60 min). (**D**) Bar graph showing Ephrin-B2/Fc stimulated COS cell migration after transfection with WT-Eph-B4 or Y774F-Eph-B4 plasmids. *P* < 0.0001 (ANOVA); *P < 0.0001 Ephrin-B2/Fc WT-Eph-B4 vs Y774F-Eph-B4. *n* = 3–4. (**E**) Representative photomicrographs (left panel) showing AVF venous wall (elastin stain) in control mice or mice treated with WT-Eph-B4 or mutant Y774F-Eph-B4. Arrow heads denote neointimal thickness. Scale bar, 25 µm. Bar graph (right panel) showing quantification of AVF venous wall thickness in control mice (white bar) or mice treated with WT-Eph-B4 (gray bar) or mutant Y774F-Eph-B4 (blue bar), day 21; *P* = 0.035 (ANOVA). *P = 0.038 (WT-Eph-B4 vs Y774F-Eph-B4; post hoc). *n* = 5–7. (**F**) Line graph showing infrarenal IVC diameter in mice with AVF treated with WT-Eph-B4 (gray line) or mutant Y774F-Eph-B4 (purple line) compared to control (black line); *P = 0.005 (ANOVA). *n* = 5–11. Data represent mean ± SEM.

To confirm a regulatory role for Eph-B4 during AVF maturation, we used Eph-B4 heterozygous mice to determine whether AVF created in mice with less Eph-B4 activity would show altered wall thickening or outward remodeling. AVF in Eph-B4 heterozygous mice had increased AVF venous wall thickening compared to that of WT mice (Fig. 3A), with a corresponding increase in proliferating cells and with no decrease in apoptotic cells (Supplementary Figure 3A and B). However, Eph-B4 heterozygous mice showed no difference in either IVC outward remodeling (Fig. 3B) or aortic outward remodeling (Supplementary Figure 3C) during fistula maturation compared to WT mice. These data confirm a regulatory role of Eph-B4 at least for venous wall thickening during AVF maturation.

We next confirmed the regulatory role of Eph-B4 during AVF maturation using a molecular approach. To determine which of the conserved fourteen intracellular tyrosines are critical to Eph-B4 function during AVF maturation, COS cells were transfected with a plasmid expressing WT murine Eph-B4 and then treated with Ephrin-B2/Fc for 3 min. LC-MS/MS identified that tyrosines 581, 590, and 774 were phosphorylated (Supplementary Figure 4A and B). Fourteen separate mutant Eph-B4 plasmids were created in which a single tyrosine residue was replaced with phenylalanine. COS cells were transfected with the individual WT- or mutant-Eph-B4 plasmids and then treated with Ephrin-B2/Fc for 3 min. Three of these mutants, Y774F, Y821F, and Y924F, showed complete absence of Eph-B4 phosphorylation with Ephrin-B2/Fc treatment (Supplementary Figure 4C). Since tyrosine 774 was identified as both an early and critical site of Eph-B4 phosphorylation, we determined whether mutation of tyrosine 774 altered Eph-B4 function *in vitro*. COS cells were transfected with WT-Eph-B4 or Y774F-Eph-B4 plasmids; the Y774F-Eph-B4 transfected cells showed a sustained lack of tyrosine phosphorylation in response to Ephrin-B2/Fc (Fig. 3C) that was not due to altered receptor localization during biotinylation of surface-expressed proteins (Supplementary Figure 4D). Similarly, cells transfected with the Y774F-Eph-B4 plasmid showed lack of caveolin-1 colocalization (Supplementary Figure 4E), reduced Ephrin-B2/Fc-stimulated migration (Fig. 3D), as well as reduced Akt and ERK1/2 phosphorylation (Supplementary Figure 4F). Since these data suggest that Eph-B4 function is dependent on tyrosine 774 activity *in vitro*, we tested the function of Eph-B4 tyrosine activity *in vivo*. Lentivirus was used to deliver HA-tagged Eph-B4 via a pluronic gel to the IVC. En face staining validated delivery of the lentivirus to the venous wall, including the endothelium (Supplementary Figure 4G). The adventitia of both the arterial and venous limbs of AVF were treated with pluronic gel containing lentivirus with WT-Eph-B4 or Y774F-Eph-B4 at the time of AVF creation. AVF treated with WT-Eph-B4 lentivirus had thin walls similar to control mice, whereas AVF treated with Y774F-Eph-B4 lentivirus had markedly thicker walls (Fig. 3E), with increased proliferating cells and no diminution in apoptotic cells (Supplementary Figure 3D and E). Interestingly, fistulae treated with WT-Eph-B4 showed less outward remodeling of both the venous limb (Fig. 3F) and the arterial limb (Supplementary Figure 3F) compared to WT mice whereas Y774F-Eph-B4 treatment did not show reduced outward remodeling. These results suggest that Eph-B4 tyrosine 774 is critical for Eph-B4 function *in vivo* during AVF maturation, and that diminished Eph-B4 function during AVF maturation promotes venous wall thickening and outward remodeling.

### Eph-B4 signaling is mediated by Akt1 *in vitro*

**Figure 4:**
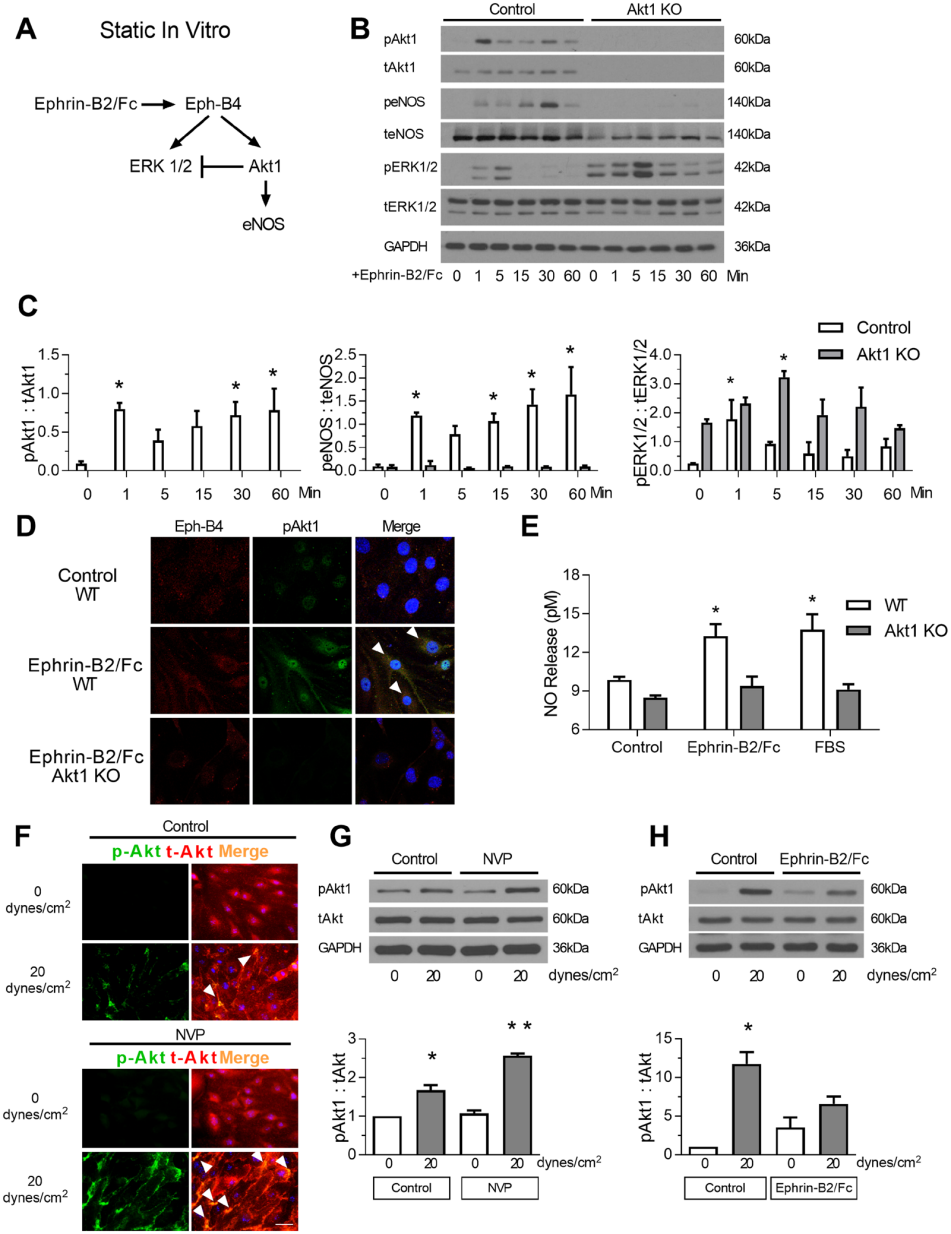
Eph-B4 signaling is mediated by Akt1 *in vitro*. (**A**) Diagram showing *in vitro* Eph-B4 and downstream signaling under static conditions. (**B**) Representative Western blot showing Ephrin-B2/Fc stimulation of Akt1, eNOS, and ERK1/2 phosphorylation in EC derived from WT or Akt1 KO mice (0–60 min). (**C**) Bar graphs show quantification of Western blot comparing EC derived from WT or Akt1 KO mice. pAkt1; *P* < 0.0001 (ANOVA). *P < 0.05 (post hoc). peNOS; *P* < 0.0001 (ANOVA). *P < 0.05 (post hoc). pERK1/2; *P* < 0.0001 (ANOVA). *P < 0.05 (post hoc). *n* = 3. (**D**) Representative photomicrographs showing immunoreactive signal to Eph-B4 (green), pAkt1 (red), and merge (yellow) in WT (top and middle row) or Akt1 KO (bottom row) EC without or with Ephrin-B2/Fc. White arrow indicates merged signal. Scale bar, 25 µm. (**E**) Bar graph showing NO release in WT or Akt1 KO EC stimulated with Ephrin-B2/Fc or FBS; *P* < 0.0001 (ANOVA). *P < 0.05 (*P* = 0.0108 WT Ephrin-B2/Fc vs control; *P* = 0.0035 WT FBS vs control; post hoc). (**F**) Representative immunofluorescence showing pAkt1 (green), tAkt1 (red), and merged (yellow) signal from WT EC under static (top row; 0 dyne/cm^2^) or flow conditions (bottom row; 20 dyne/cm^2^) without (upper panel) and with the Eph-B4 inhibitor NVP-BHG712 (lower panel). White arrowheads indicates colocalized immunoreactivity. (**G**) Western blot (upper panel) and accompanying bar graph (lower panel) showing pAkt1 expression stimulated by laminar shear stress, without and with pretreatment with the Eph-B4 inhibitor NVP-BHG712; *P* < 0.0001 (ANOVA). *P = 0.0018 (post hoc); **P = 0.0003 (post hoc); *n* = 3. (**H**) Western blot (upper panel) and accompanying bar graph (lower panel) showing pAkt1 expression stimulated by laminar shear stress, without and with pretreatment with the Ephrin-B2/Fc; *P* = 0.0094 (ANOVA). *P = 0.0083 (post hoc); *n* = 2. Data represent mean ± SEM.

Since Eph-B4 stimulates Akt phosphorylation *in vitro* and Akt is a critical factor promoting endothelial cell survival and function^17^, we examined whether Akt is a critical factor that mediates Eph-B4 function in an *in vitro* static environment (Fig. 4A **)**. Endothelial cells (EC) derived from WT and Akt1 KO mice were stimulated with Ephrin-B2/Fc; as expected, WT EC showed increased Akt1, eNOS, and ERK1/2 phosphorylation; Akt1 KO EC did not show Akt1 or eNOS phosphorylation but had increased ERK1/2 phosphorylation (Fig. 4B and C). In WT EC, Akt1 phosphorylation colocalized with Eph-B4 in the cytoplasm after Ephrin-B2/Fc stimulation (Fig. 4D, white arrowheads). Similarly, Ephrin-B2/Fc stimulated NO release in WT cells that was inhibited in Akt1 KO EC (Fig. 4E), suggesting that Akt1 regulates some Eph-B4 functions in EC *in vitro*.

We next determined if Eph-B4 similarly stimulates Akt1 phosphorylation in an *in vitro* flow environment. Compared to the basal amount of Akt1 phosphorylation in EC under static conditions (0 dynes/cm^2^), there was increased Akt1 phosphorylation in response to arterial magnitudes of laminar shear stress (20 dynes/cm^2^); however, pretreatment of EC with the Eph-B4 inhibitor NVP-BHG712 resulted in further increased Akt1 phosphorylation (Fig. 4F and G). Conversely, pretreatment of EC with Ephrin-B2/Fc to activate Eph-B4 resulted in decreased shear stress inducted Akt1 phosphorylation (Fig. 4H **)**. These results suggest that Eph-B4 inhibits laminar shear stress-induced Akt1 phosphorylation, e.g. Akt1 mediates some functions of Eph-B4 in EC *in vitro*, under both static and flow conditions, but the effect of Eph-B4 on Akt1 activity *in vitro* may differ under static and flow conditions.

### Akt1 mediates venous remodeling *in vivo*

**Figure 5:**
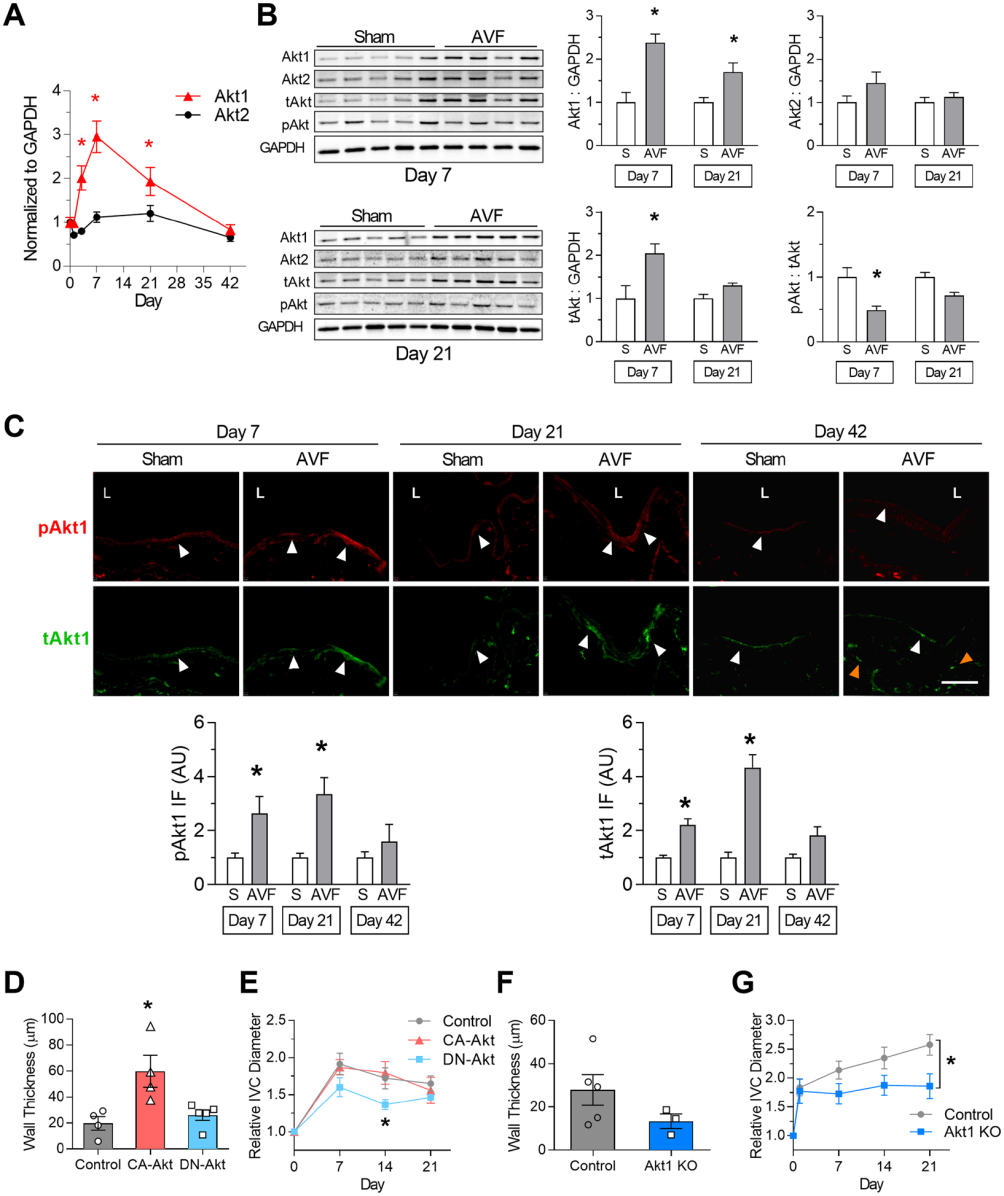
Akt1 mediates venous remodeling *in vivo*. (**A**) Line graph showing *Akt1* and *Akt2* mRNA expression in venous limb of AVF; *P* < 0.0001 (ANOVA). *P < 0.05 (*P* = 0.0127 at day 3; *P* < 0.0001 at day 7; *P* = 0.0289 at day 21; post hoc). *n* = 7–8. (**B**) Representative Western blots (left hand panels) showing Akt1, Akt2, tAkt, and pAkt expression in sham veins and venous limb of AVF. Bar graphs (right hand panels) show quantification. Akt1: *P* < 0.0001 (ANOVA). *P < 0.05 (*P* = 0.0005 at day 7; *P* = 0.0415 at day 21; post hoc). Akt2: *P* = 0.08 (ANOVA). tAkt: *P* = 0.0032 (ANOVA). *P < 0.05 (*P* = 0.0039 at day 7; post hoc). pAkt:tAkt ratio: *P* = 0.0007 (ANOVA). *P < 0.05 (*P* = 0.0041 at day 7; post hoc). *n* = 4–5. (**C**) Representative immunofluorescence (upper panels) showing pAkt1 (red) and tAkt1 (green) signal in sham veins or AVF, day 7 (left columns), day 21 (middle columns), or day 42 (right columns). White arrowheads denote positive signals in endothelium; orange arrowheads denote positive signals in subintima. L = vessel lumen. Bar graphs (lower panel) show quantification of immunoreactive signal. pAkt1; *P* = 0.0030 (ANOVA). *P < 0.05 (*P* = 0.0422 at day 7; *P* = 0.0339 at day 21; post hoc). tAkt1; *P* < 0.0001 (ANOVA). *P < 0.05 (*P* = 0.0214 at day 7; *P* = 0.0002 at day 21; post hoc). *n* = 3–4. Scale bar, 100 µm. (**D**) Bar graph showing AVF venous limb wall thickness in WT mice treated with control adenovirus (gray bar), CA-Akt adenovirus (red bar), or DN-Akt adenovirus (blue bar) (day 21); *P* = 0.0095 (ANOVA). *P < 0.05 (*P* = 0.0121 CA-Akt vs control; *P* = 0.0232 CA-Akt vs DN-Akt; post hoc). *n* = 4–5. (**E**) Line graph showing infrarenal IVC diameter in WT mice treated with control adenovirus (gray line), CA-Akt adenovirus (red line), or DN-Akt adenovirus (blue line); *P* = 0.0068 (ANOVA). *P < 0.05 (*P* = 0.0458 at day 14, DN-Akt vs control; *P* = 0.0138 at day 14, DN-Akt vs CA-Akt; post hoc). *n* = 4–5. (**F**) Bar graph showing AVF wall thickness in WT and Akt1 KO mice (day 21); *P* = 0.19 (t test). *n* = 3–5. (**G**) Line graph showing infrarenal IVC diameter in WT (gray) and Akt1 KO mice (blue); *P = 0.0012 (ANOVA). *n* = 8–14. Data represent mean ± SEM.

Since Akt1 may mediate some functions of Eph-B4 in EC *in vitro*, we determined if Akt1 expression is regulated during AVF maturation. *Akt1* mRNA expression increased during AVF maturation, with maximum expression at day 7; there was no difference in *Akt2* mRNA expression (Fig. 5A). Similarly, there was increased Akt1 protein expression with no significant change in Akt2 expression at days 7 and 21 (Fig. 5B). Immunofluorescence showed increased Akt1 immunoreactivity predominantly in the AVF endothelium at days 7 and 21; at day 42 there was less endothelial Akt1 immunoreactivity, although there was sustained Akt1 expression in the AVF wall, below the neointima (*P* = 0.0314; Fig. 5C). These data show that Akt1 expression is regulated during AVF maturation, in a time course similar to Eph-B4 expression; therefore, we determined if altered Akt1 activity directly affects AVF maturation. AVF in WT mice were treated with an adenovirus containing either constitutively active Akt1, dominant negative Akt1, or control virus. AVF treated with constitutively active Akt1 showed increased venous wall thickening compared to AVF treated with control or dominant negative Akt1 virus (Fig. 5D). AVF treated with constitutively active Akt1 showed similar outward remodeling compared to AVF treated with control virus, but AVF treated with dominant negative Akt1 showed reduced outward remodeling (Fig. 5E); there was no difference in arterial remodeling in any of these groups (Supplementary Figure 5A–B). Similarly, there was increased eNOS activity in CA-Akt treated mice, similar to the increased activity in AVF in WT mice, which was not present in mice treated with DN-Akt (Supplementary Figure 5C,D, and E). These results suggest that Akt1 signaling has a functional effect during AVF maturation, and that Akt1 activity may mediate venous remodeling. To determine if Akt1 mediates venous remodeling, AVF were performed in Akt1 KO and WT mice. There was a trend toward reduced venous wall thickening (Fig. 5F) in Akt1 KO mice compared to WT mice. In addition, there was reduced venous outward remodeling in Akt1 KO mice (Fig. 5G). These results support a mechanistic role for Akt1 during adaptive venous remodeling.

### Eph-B4-mediated venous remodeling depends on Akt1

**Figure 6:**
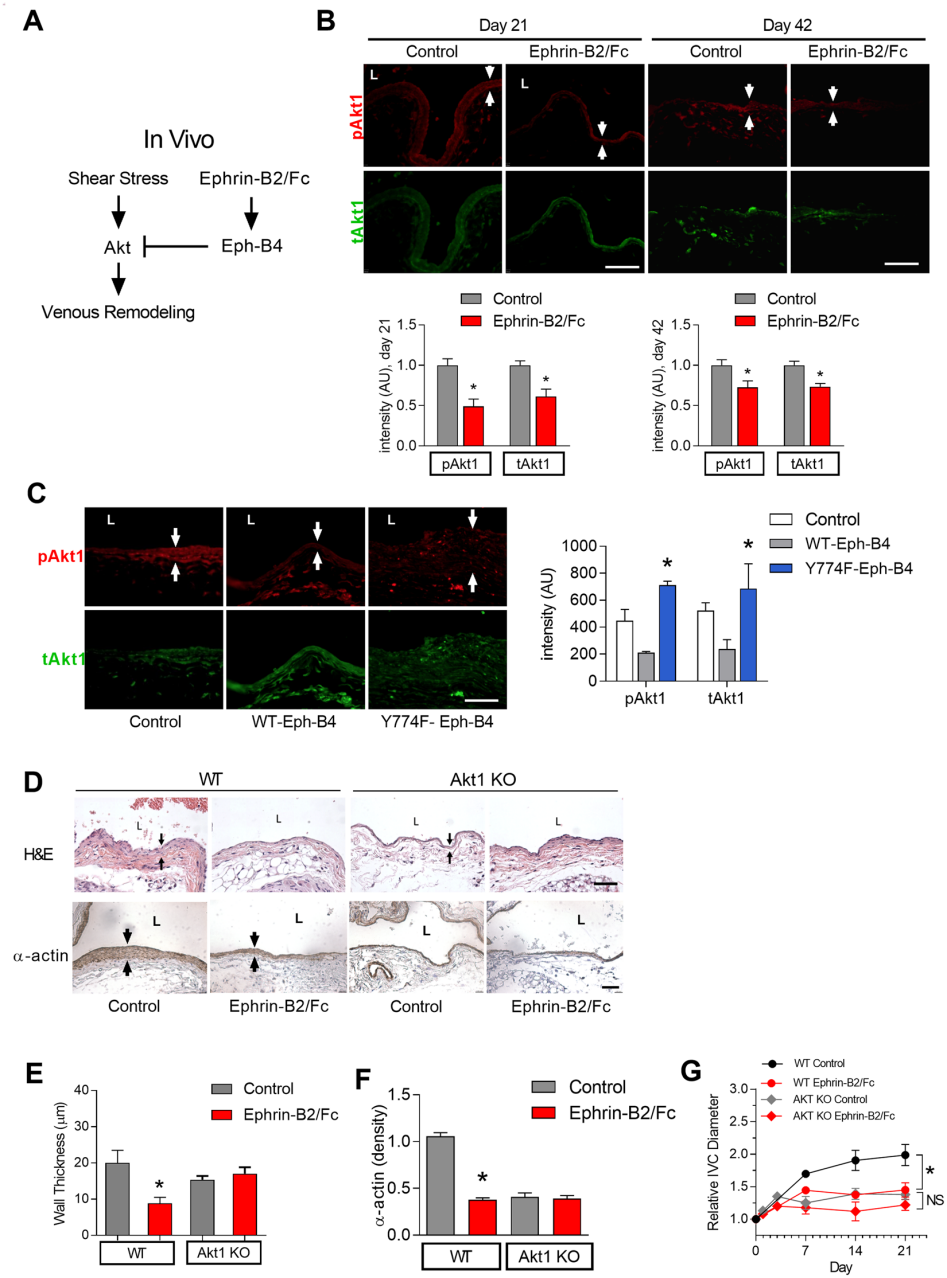
Eph-B4-mediated venous remodeling depends on Akt1. (**A**) Diagram showing *in vivo* Eph-B4 signaling inhibits shear stress activation of Akt to inhibit AVF wall thickening. (**B**) Representative immunofluorescence (upper panels) showing pAkt1 (red) or tAkt (green) in the AVF of WT mice treated with control or Ephrin-B2/Fc (day 21, left panel; day 42, right panel). White arrows show the AVF wall. Scale bar, 100 µm. Bar graphs (lower panel) show quantification. pAkt1; *P = 0.0013 at day 21, *P* = 0.0343 at day 42 (t-test). tAkt1; *P = 0.0180 at day 21, *P* = 0.0022 at day 42 (t-test). *n* = 3 for day 21; *n* = 5–7 for day 42. (**C**) Representative immunofluorescence (left panel) showing pAkt1 (red) or tAkt1 (green) in the AVF of WT mice treated with wild type Eph-B4 lentivirus (WT-Eph-B4) or mutant Y774F-Eph-B4 lentivirus (Y774F-Eph-B4) compared to control mice (day 21). Scale bar, 100 µm. Bar graph (right panel) shows quantification. *P* < 0.0001 (ANOVA). *P < 0.05 (*P* = 0.0051 pAkt1 WT-Eph-B4 vs Y774F-Eph-B4; *P* = 0.010 tAkt1 WT-Eph-B4 vs Y774F-Eph-B4; post hoc). *n* = 3. (**D**) Photomicrographs showing H&E staining (top row) and immunohistochemistry for α-actin (bottom row) in the wall of WT (first 2 columns) and Akt1 KO mice (last 2 columns) without and with Ephrin-B2/Fc treatment (day 21). Black arrows show the AVF wall. L = vessel lumen. Scale bar, 50 µm. (**E**) Bar graph showing AVF venous limb wall thickness in WT and Akt KO mice without and with Ephrin-B2/Fc stimulation (day 21); P = 0.0318 (ANOVA). *P = 0.0234 (WT control vs Ephrin-B2/Fc; post hoc). *n* = 3–4. (**F**) Bar graph showing quantification of α-actin density in the wall of AVF in WT and Akt1 KO mice without or with Ephrin-B2/Fc (day 21); P < 0.0001 (ANOVA). *P < 0.0001 (WT control vs Ephrin-B2/Fc; post hoc). n = 3. (**G**) Line graph showing infrarenal IVC diameter in WT and Akt1 KO mice without or with Ephrin-B2/Fc; *P < 0.0001 (ANOVA, WT); NS, *P* = 0.137 (ANOVA, Akt1 KO). *n* = 3–5. Data represent mean ± SEM.

Since Eph-B4 regulates venous wall thickening and outward remodeling, and Akt1 also mediates venous wall thickening and outward remodeling, we determined if Akt1 is a mechanism by which Eph-B4 regulates adaptive venous remodeling during AVF maturation (Fig. 6A). AVF in WT mice treated with Ephrin-B2/Fc showed reduced phosphorylated Akt1 immunoreactivity as well as less total Akt1 immunoreactivity compared to control AVF (Fig. 6B), suggesting that Eph-B activation inhibits Akt1 expression during venous remodeling *in vivo*. To confirm that Eph-B4 inhibits Akt expression during venous remodeling, AVF were treated with WT-Eph-B4 or mutant Y774F-Eph-B4 lentivirus. AVF in WT mice treated with mutant Y774F-Eph-B4 lentivirus showed increased Akt1 phosphorylation and total Akt immunoreactivity compared to mice treated with WT-Eph-B4 lentivirus (Fig. 6C). These results are consistent with Eph-B4 inhibiting Akt1 function during venous remodeling *in vivo*.

To directly test whether Akt1 mediates Eph-B4 regulated venous remodeling, Eph-B4 function was stimulated with Ephrin-B2/Fc after AVF creation in WT and Akt1 KO mice. In WT mice, Ephrin-B2/Fc treatment was associated with thin venous walls (Fig. 2) with less α-actin compared to control veins that were thick and had more α-actin; however, veins of Akt1 KO mice treated with Ephrin-B2/Fc did not have thinner walls nor less actin staining compared to control Akt1 KO veins (Fig. 6D–F). Similarly, WT mice treated with Ephrin-B2/Fc had less venous outward remodeling compared to WT mice (Fig. 2), whereas Akt1 KO mice treated with Ephrin-B2/Fc did not show less venous outward remodeling (Fig. 6G). In toto, these data show that Eph-B4 inhibits Akt1 phosphorylation and expression during AVF maturation, and that reduced Eph-B4 function during normal AVF maturation is associated with increased Akt1 function, suggesting that Eph-B4 inhibits Akt1-mediated venous remodeling.

**Figure 7:**
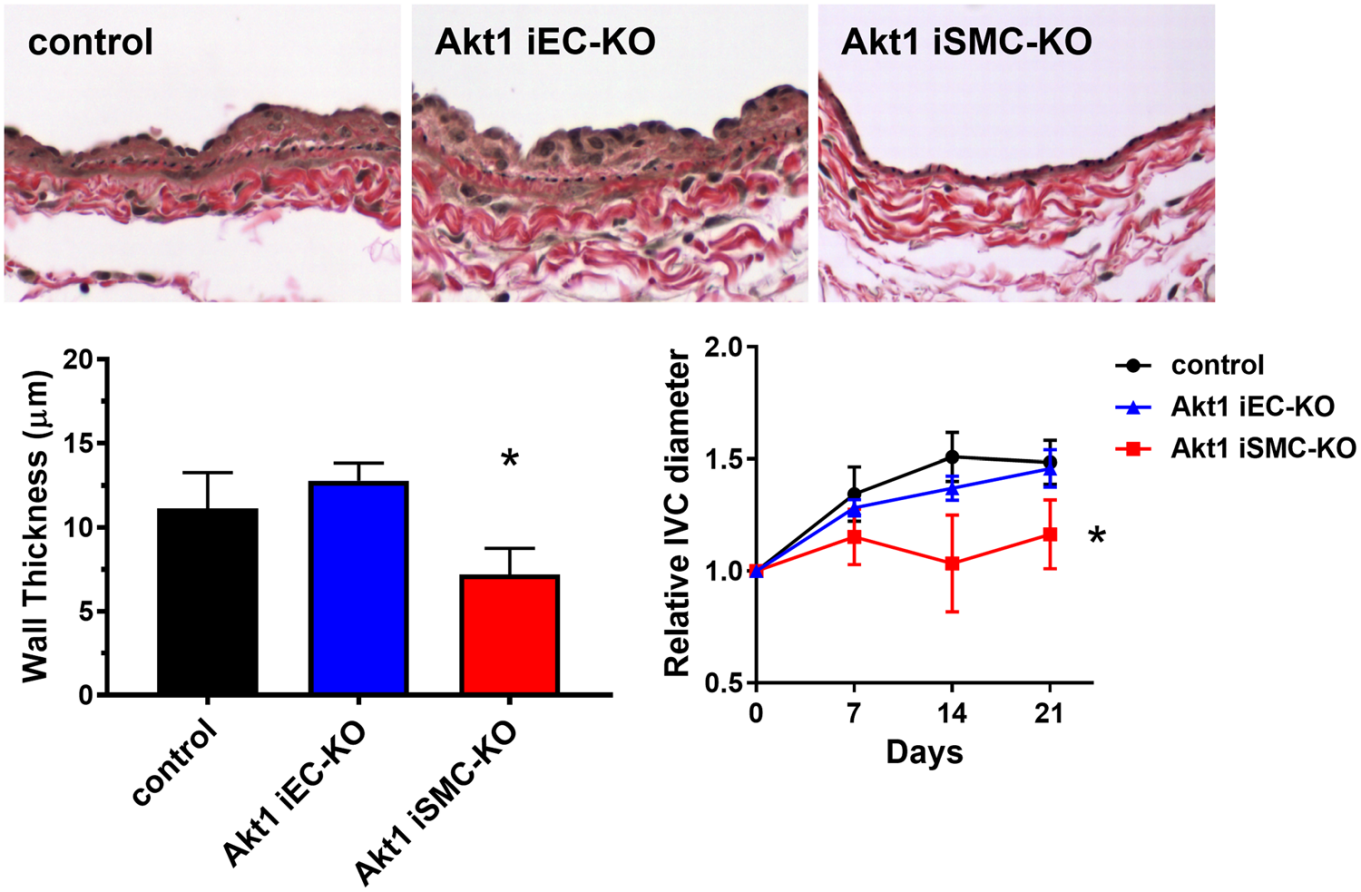
Venous remodeling depends on smooth muscle cell Akt1. (**A**) Representative photomicrographs showing the venous wall (day 21) after AVF creation in control, Akt1 iEC-KO, and Akt1 iSMC-KO mice. (**B**) Bar graph of AVF wall thickness. *P = 0.0146 compared to Akt1 iEC-KO, t-test. N = 8 for control; N = 6 for Akt1 iEC-KO; N = 4 for Akt1 iSMC-KO. (**C**) Relative diameter of the AVF over time after AVF creation in control, Akt1 iEC-KO, and Akt1 iSMC-KO mice. *P = 0.0046, ANOVA. N = 9 for control; N = 5 for Akt1 iEC-KO; N = 5 for Akt1 iSMC-KO.

We have previously shown, using selective knockdown of Akt1 from endothelial cells, that Akt1 is critical for angiogenesis^23^. AVF created in mice that underwent tamoxifen-inducible Akt1 deletion in endothelial cells showed similar wall thickness and dilation (21 days) compared to control mice that received vehicle alone (Fig. 7). However, AVF created in mice that underwent tamoxifen-inducible Akt1 deletion in smooth muscle cells showed reduced wall thickness and dilation (21 days) compared to control mice (Fig. 7). These results are consistent with smooth muscle cell Akt1 playing a critical role in venous remodeling in the fistula environment.

## Discussion

We report that expression of the Eph-B4 receptor as well as its ligand Ephrin-B2 increase in the vein wall during AVF maturation (Fig. 1), consistent with acquisition of dual arterial-venous identity in the fistula environment; increased Eph-B activity regulates venous remodeling, leading to improved long-term patency (Fig. 2). Directed point mutations of the Eph-B4 receptor identified cytoplasmic tyrosine 774 as a critical phosphorylation site for Eph-B4 function and delivery of mutant Eph-B4 receptors with a nonfunctional tyrosine 774 resulted in altered venous remodeling, similar to that seen in heterozygous Eph-B4 mice (Fig. 3). *In vitro*, Eph-B4 inhibits shear stress-induced Akt1 phosphorylation, a different effect on Akt1 phosphorylation observed under static conditions (Fig. 4). *In vivo*, Akt1 is a critical mediator of venous remodeling (Fig. 5), particularly smooth muscle cell Akt1 (Fig. 7), and Akt1 is a mechanism of Eph-B-mediated venous remodeling (Fig. 6). In toto, these data show that veins adapt to the fistula environment by acquisition of dual arterial-venous identity, whereas veins adapt to the arterial environment by loss of vessel identity^7,20^; in addition these data show that Eph-B4 regulates venous remodeling by an Akt1-mediated mechanism.

Our data showing that Eph-B4 regulates venous remodeling during AVF maturation are consistent with previous reports showing that Eph-B4 inhibits venous remodeling during vein graft adaptation^19,20^. Interestingly, the expression pattern of Eph-B4 and Ephrin-B2 is different between the AVF and vein graft models. We previously showed that vein graft adaptation is characterized by loss of venous identity, with reduced Eph-B4 expression, but without a gain of arterial identity^7,20^. Here we report that during AVF maturation the vein acquires dual vessel identity, with increased venous and arterial markers (Fig. 1, Table 1). We verified this finding using an alternative model by placing a patch into a rat IVC, without or with a fistula, to confirm that the presence of a fistula increases both Eph-B4 and Ephrin-B2 expression (Fig. 1). Acquisition of dual arterial and venous identity has not been previously described during physiological venous adaptation such as occurs during AVF maturation; increased expression of both Eph-B4 and Ephrin-B2 has been reported in some cancers^24–27^. The different gain of dual arterial-venous identity during AVF maturation that is distinct from the loss of vessel identity during vein graft adaptation demonstrates the distinct biology of AVF maturation in the fistula environment from that of vein graft adaptation to the arterial environment; these molecular differences correlate with the distinct clinical outcomes of these two procedures, with AVF associated with distinctly worse outcomes compared to vein grafts^12,28^. However, the differential mechanisms by which vessel identity is regulated in these two environments is not known. Notably, our data regarding vessel identity (Table 1 and Fig. 1) is derived from the whole AVF wall; it is not clear whether different cell components or layers within the vessel have different identities or are differentially altered during vein graft adaptation or AVF maturation, and thus could be mechanistic or simply markers of these processes.

There are numerous differences between the arterial and fistula environments, including differences in flow, pressure, resistance of the runoff bed, oxygen tension, and regular environmental injury^10^. Hemodynamics can regulate Ephrin-Eph signaling and vessel function^29^; for example, laminar shear stress can reduce Ephrin-B2 and Eph-B4 expression^30–32^, and Ephrin-B2-Eph-B4 interactions promote monocyte adhesion to the endothelium under flow conditions^33^. Ephrin-Eph signaling is also involved in blood pressure regulation^34^, and stretch-induced Ephrin-B2 expression limits smooth muscle cell migration and monocyte extravasation, promoting arteriogenesis^35^. Other mechanisms may also be functional, as endothelial Ephrin-B2 also regulates vasodilation and NO signaling^36^. Since both Ephrin-B2 and Eph-B4 expression increase during AVF maturation (Fig. 1), it is likely that numerous factors regulate EphrinB-EphB signaling *in vivo*, and that EphrinB-EphB signaling is only a single component of this complex adaptive process.

Using the w804a point mutation in the Eph-B4 caveolin-binding domain, we previously showed that Eph-B4 phosphorylation is critical to its function; however, in that study we did not identify the critical tyrosine that is responsible for the activity of the Eph-B4 kinase domain^20^. Here we used mass spectrometry to determine that three Eph-B4 intracellular tyrosines, Y581, Y590, and Y774, are phosphorylated within 3 min of stimulation with the Eph-B4 ligand Ephrin-B2/Fc; this pattern is consistent with canonical Eph receptor activation^13^. Methodical mutation of all Eph-B4 intracellular tyrosines showed that only Y774 was both phosphorylated within 3 minutes of activation and was also critical for Eph-B4 receptor activity (Supplementary Figure 4). Eph-B4 tyrosine 774 is critical for Eph-B4 receptor tyrosine phosphorylation, Akt and ERK1/2 phosphorylation (Supplementary Figure 4), and cell migration *in vitro* (Fig. 3), as well as venous remodeling *in vivo* (Fig. 3), e.g. tyrosine 774 is a critical mediator of Eph-B4 function both *in vitro* and *in vivo*. It is possible that the effects of Eph-B4-Y774F are pleotropic among all the cell types of the vessel wall, and that overexpressed Eph-B4 may act as a sink for Ephrin-B2 ligands, altering multiple forward and reversed signaling pathways. However, in toto, these results are consistent with previous reports showing the importance of tyrosine phosphorylation for activation and intracellular signaling of Eph-B receptors^13^, and complement our data using Ephrin-B2/Fc as an activator of Eph-B signaling (Figs 2, 4, 6) as well as our data using heterozygous Eph-B4 mice with reduced Eph-B4 signaling (Fig. 3). Nonetheless, it is possible that Eph-B receptors on the many components of the vessel wall, including endothelial, smooth muscle, mesenchymal and immune cells, alters AVF remodeling, and this is consistent with our finding that smooth muscle Akt1 is critical for venous remodeling (Fig. 7). Acquisition of dual arterial-venous identity during AVF maturation, e.g. colocalization of cells expressing EphrinB ligands with cells expressing EphB receptors, may be an important mechanism of venous remodeling; however, the relative contributions of EphrinB- and EphB- expressing cells that are native cell components of the vessel wall, or infiltrating cells such as inflammatory cells or M2 macrophages^37^, is not clear.

Our data also show that Akt1 function is critical for venous remodeling that occurs during AVF maturation and is a mechanism of Eph-B4 regulation of venous remodeling. Akt promotes cell survival, protein synthesis, and growth that are necessary for adaptive remodeling of the vein to the fistula environment. VEGF activates the Akt-eNOS pathway, while inhibiting ERK1/2, to promote maintenance of the vascular system^38^; similarly Eph-B4 activates the Akt-eNOS pathway both *in vitro and in vivo* ^17,21^. Interestingly, Akt expression is increased during the venous remodeling that occurs during vein graft adaptation, but under these different hemodynamic conditions neither Akt nor eNOS is phosphorylated^19^. However, it is not clear why Akt is activated during venous remodeling in the fistula environment whereas Akt is not activated during venous remodeling in the arterial environment. We also observed that Eph-B4 stimulates Akt1 phosphorylation under static conditions but inhibits Akt1 phosphorylation under laminar shear stress conditions *in vitro* (Fig. 4), suggesting that Eph-B4 may have mechanosensory functions. Nevertheless, our data shows that loss of Akt1 function abolishes Eph-B4 regulation of venous remodeling (Fig. 6), suggesting that Akt1 is a mechanism of Eph-B4-mediated venous remodeling such as occurs during AVF maturation *in vivo*. Interestingly, selective deletion of Akt1 from smooth muscle cells, but not endothelial cells, abolished venous remodeling (Fig. 7); this data is consistent with the recent description of differentiated smooth muscle cells contributing to medial wall thickening during AVF maturation^39^. This data also confirms the critical importance of Akt1 to venous remodeling during AVF maturation.

In summary, we show that Eph-B4 expression increases during AVF maturation and that Eph-B4 activity, mediated by tyrosine 774, regulates adaptive venous remodeling through an Akt1-mediated mechanism. Stimulation of Eph-B4 attenuated Akt1 expression i*n vivo*, leading to improved long-term patency in this animal model. Eph-B4 is normally present in adult veins and may be a potential therapeutic target to reduce pathologic venous remodeling that is associated with failure of fistula maturation. These results suggest that strategies to manipulate vessel identity by altering Eph-B4 activity may improve AVF maturation.

## Methods

### Human specimens

The principles outlined in the Declaration of Helsinki were followed, and approval of the Veterans Affairs Human Investigation Committee was obtained. Deidentified discarded specimens of vein and AVF were obtained during revision operations of functional AVF; informed consent to use the samples was obtained.

### Infrarenal aorto-caval fistula

All animal experiments were performed in strict compliance with federal guidelines and with approval from the Yale University IACUC. Mice used for this study included wild type C57BL6/J (WT), Eph-B4 heterozygous (Eph-B4 het)^20^, or Akt1 knockout (Akt1 KO) mice^40^. Smooth muscle-specific knockouts were generated by breeding Akt1^flox/flox^ with the Myh11-CreERT2 strain^23^. Deletion of Akt1 was induced by injecting 4–5 week old mice with tamoxifen (100 ug/g total body weight) for 5 consecutive days. Akt1^flox/flox^ control mice were similarly injected with vehicle. The Akt1^flox/flox^ were bred to the Cdh5-Cre-ERT2 mice to obtain inducible, endothelial-targeted Akt1 mice, as previously described^23^. Young adult, male mice (~4–5 wks of age) were injected with tamoxifen (100 ug/g total body weight) via intraperitoneal delivery for 7 consecutive days. Phenotyping assessments were performed 6 weeks post-tamoxifen administration (~10–12 wks of age).

All mice were male and 9–12 weeks of age when the infrarenal aorto-caval fistulae were created as previously described^22,41^. Briefly, AVF were created by needle puncture from the aorta into the IVC. Visualization of pulsatile arterial blood flow in the IVC was assessed as a technically successful AVF.

### Measurement of fistula dilation *in vivo*

Doppler ultrasound (40 MHz; Vevo770 High Resolution Imaging System; VisualSonics Inc., Toronto, Ontario, Canada) was used to confirm the presence of the AVF and to measure the diameter of the vessels as previously described^22,41^. Doppler ultrasound was performed the day prior to operation (pre-op values) and serially post-operatively. Increased diastolic flow through the aorta and a high velocity pulsatile flow within the IVC were used to confirm the presence of an AVF during post-operative interrogations, and AVF that were not patent on Doppler study were explanted; patency was confirmed at AVF explantation by direct visualization of pulsatile arterial blood flow in the IVC, and in all cases correlated with the ultrasound findings.

### Histology

After euthanasia, the circulatory system was flushed under pressure with PBS followed by 10% formalin and the AVF was extracted en bloc. The tissue was then embedded in paraffin and cut in 5 μm cross sections. Hematoxylin and eosin (H&E) staining was performed for all samples. For wall thickness measurements, cross sections were obtained 125 μm cranial to the AVF and stained with elastin van Gieson (EVG) stain. Eight equidistant points per cross section were averaged to obtain the mean outer wall diameter encompassing the intima and media, as previously described^7,20^. Additional unstained cross sections in this same region (100–150 μm cranial to the AVF) were used for immunohistochemical or immunofluorescence microscopy.

### Immunohistochemistry

Sections were heated in citric acid buffer (pH 6.0) at 100 °C for 10 min for antigen retrieval. Sections were then treated with 0.3% hydrogen for 30 min and blocked with 5% normal goat serum containing 0.05% Triton X‐100 (T‐PBS). Sections were then incubated overnight at 4 °C with the following primary antibodies diluted in T-PBS: anti-Eph-B4 (Abcam, ab64820), anti-Ephrin-B2 (Novus, NBP1–49857), anti-Ki67 (Abcam, ab15580), or anti-cleaved caspase-3 (cell signaling, #9661).

### Immunofluorescence

Antigen retrieval was done in the same manner as IHC described above. After pretreatment, sections were blocked with 5% normal goat serum in T‐PBS for 1hr at room temperature and then incubated overnight at 4 °C with the following primary antibodies diluted in T-PBS: anti-Ephrin-B2 (Santa Cruz, SC1010 or SC19227), anti-Eph-B4 (R&D, AF446 or Santa Cruz, SC5536), anti-vWF (Abcam, ab1713), anti-alpha-actin (Abcam, ab5694), anti-phospho-tyrosine (Abcam, ab10321), anti-phospho-Akt1 (Cell Signaling, #9018), or anti-Akt1 (Cell Signaling, #2967). Sections were then treated with secondary antibodies at room temperature for 1–2 hr using goat anti-rabbit Alexa Fluor 488 (Life Technologies, Grand Island, NY), donkey anti-goat Alexa-Fluor-488 (Life Technologies), or donkey anti-rabbit Alexa-Fluor-568 (Life Technologies). Sections were stained with SlowFade® Gold Antifade Mountant with DAPI (Life Technologies) and coverslip applied. Digital fluorescence images were captured and intensity of immunoreactive signal was measured using Image J software (NIH, Bethesda, Maryland). Intensity of merge signal was determined by applying a color threshold selective for yellow signal.

### Western Blot

The venous limb of the AVF was harvested and treated with RIPA lysis buffer containing protease inhibitors. Equal amounts of protein were loaded and run in SDS-PAGE followed by Western blot analysis. Protein expression was probed with the following antibodies: Eph-B4 (R&D, AF446), Ephrin-B2 (abcam, ab75868), pan-Akt (Cell Signaling, #4685), total-Akt1 (Cell Signaling, #2938), or total-Akt2 (Cell Signaling, #3063). Membrane signals were detected using ECL detection reagent (GE Healthcare, Denville scientific) or the LI-COR Odyssey imaging system (Lincoln, NE).

### RNA extraction and quantitative PCR

Total RNA from the venous limb of the AVF was isolated using the RNeasy Mini kit. Reverse transcription was performed using the SuperScript III First‐Strand Synthesis Supermix (Invitrogen, Carlsbad, CA). Real‐time quantitative PCR was performed using SYBR Green Supermix (Bio‐Rad Laboratories, Hercules, CA) and amplified for 40 cycles using the iQ5 Real‐Time PCR Detection System (Bio‐Rad Laboratories). Correct target amplification and exclusion of nonspecific amplification was confirmed by 1.5% agarose gel electrophoresis, and primer efficiencies were determined by melt curve analysis. All samples were normalized by *GAPDH* RNA amplification.

### Rat venous patch model

All animal experiments were performed in strict compliance with federal guidelines and with approval from the Yale University IACUC. 6–8 week old male Wistar rats were used for patch implantation as previously described^21^. Rats were divided into three groups and underwent bovine pericardial patch (LeMaitre Vascular, Burlington, MA) implantation into the IVC, the aorta, or the IVC in the presence of an aorto-caval fistula (IVC + AVF). For the IVC + AVF group, after the patch was implanted into the IVC, the distal micro-clamp of the IVC was removed first to flush out the air, then the proximal micro-clamp was removed, and IVC flow was restored for 5 minutes. Next, the aorta was exposed and the infrarenal aorta was clamped. A 20-gauge needle was used to puncture through the aorta into the adjacent IVC just above the aorta bifurcation. 10–0 suture was used to close the puncture site on the aorta and the aortic clamp was then removed. The abdomen was closed using 5–0 Dacron sutures. Animals were sacrificed on postoperative day 14 for histology with IHC and IF as described above. No immunosuppressive agents, antibiotics or heparin were given at any time.

### Eph-B4 stimulation *in vivo*

Eph-B4 was stimulated with Ephrin-B2/Fc (R&D, Minneapolis, MN). 24 hr prior to AVF creation, mice underwent ultrasound for measurement of preoperative vessel diameter as described above. While still under general isoflurane anesthesia, 20 µg of Ephrin-B2/Fc diluted in 200 µL PBS was delivered by intraperitoneal injection (IP). Control mice received an equal volume injection of vehicle (PBS) as control. The next day, an infrarenal aorto-caval AVF was created as described above. Additional IP injections of Ephrin-B2/Fc were delivered every 48 hr beginning on postoperative day 1 and continued throughout the study period.

### Cell Culture

Cos-7 (COS) cells were passaged at 80–90% confluence on high glucose DMEM (Dulbecco’s modified Eagle’s medium, Gibco Life Technologies) supplemented with 10% fetal bovine serum (FBS, Gibco Life Technologies), L-glutamine, and penicillin/streptomycin. Mouse lung endothelial cells (EC) were prepared as described previously^23^ and were cultured in EBM-2 (Endothelial Basal Media-2) supplemented with 20% FBS, L-glutamine, penicillin/streptomycin, and EGM-2 Bulletkit (Lonza).

### Plasmid transfection

Cells were plated on 6-well plates at a concentration of 300,000 cells per well. The following day, cells were treated with plasmid and Lipofectamine 2000 (Life Technologies) diluted in OptiMEM (Life Technologies). Plates were incubated for 4–5 hr at 37 °C, 5% CO_2_. The media was then replaced with complete media and incubated overnight at 37 °C, 5% CO_2_. The following day the media was aspirated and the cells starved for 24 hr by the addition of 2 ml of OptiMEM per well.

### Eph-B4 stimulation *in vitro*

Following starvation, cells were stimulated with either mouse Ephrin-B2/Fc (2 µg/ml) or mouse IgG/Fc (2 µg/ml; R&D Systems) in PBS, for the designated amount of time, after which either the cell lysates or the conditioned media were collected.

### *In vitro* shear stress

EC were cultured to confluence on collagen I-coated glass plates (StreamerTM Culture Slips, Flexcell Corporation). Confluent EC were serum-starved for 24 hr and then pre-treated with NVP-BHG712 (1 µM; Sigma Aldrich, St. Louis, MO) for 1 hr or stimulated with 2 µg/ml mouse Ephrin-B2/Fc (R&D Systems) for 1 hr and compared to control. After pre-treatment (37 °C, 5% CO_2_), cells were exposed to steady laminar flow in a parallel-plate flow chamber with circulating EBM-2 medium (0% FBS, 37 ± 0.5 °C, 1 hr). Wall shear stress was calculated by the formula τ = 6 μQ/bh^2^, where μ is the viscosity of the fluid, Q is the flow volume (ml/s), b is the width of the flow channel in cm, and h is the height of the flow channel in cm; shear stress was set at 0 dynes/cm^2^ or 20 dynes/cm^2^. After shear stress treatment, the EC on the slides were removed with RIPA lysis buffer and analyzed.

### *In vitro* whole cell lysate isolation

Cells were treated with RIPA lysis buffer containing protease and phosphatase inhibitor cocktail. Cells were then scraped, sonicated and centrifuged.

### Mutagenesis of Eph-B4 tyrosine residues

The cDNA sequence of mouse Eph-B4 was purchased from Open Biosystems (GE Dharmacon, Lafayette, CO) and corresponds to transcript variant 2 (NCBI Reference Sequence: NM_010144.6). The cDNA was amplified using appropriate primers. The PCR product was prepared for insertion into the pShuttle-IRES-hrGFP-2 plasmid vector (Agilent Technologies, Santa Clara, CA) by digestion with *SpeI* and *PvuI*. The QuikChange Site-Directed Mutagenesis Kit (Agilent) was utilized for generating phenylalanine substitutions for cytoplasmic tyrosine residues. Sense and antisense mutant primers were designed using PrimerX (www.bioinformatics.org/primerx). After amplification with thermal cycling the product was digested with *Dpn* I for 1 hr at 37 °C and then transformed into XL10-Gold ultracompetent cells with the addition of β-mercaptoethanol through heat shocking at 42 °C for 30 sec. Under sterile conditions the transformed cells were spread onto agar plates and a miniprep spin kit (Qiagen) was used for the purification and isolation of plasmid DNA. Newly formed plasmid samples underwent template sequencing to confirm the substitution of phenylalanine for tyrosine at the desired site.

### Identification of phosphorylated Eph-B4 tyrosine residues

COS cells were transfected with 1 µg of HA-tagged mouse wt-Eph-B4. After 24 hr starvation, the transfected COS cells were treated with Ephrin-B2/Fc (2 μg/ml) for 1 min. The whole cell lysate was collected as described above, and then underwent immunoprecipitation for the HA-tag using a sepharose bead conjugate (Cell Signaling). Immunoprecipitation samples underwent polyacrylamide gel electrophoresis using a pre-cast 10% SDS-PAGE gel (BioRad). All equipment underwent three methanol rinse and washes. Nitrile gloves were used at all times when handling samples. After completion of electrophoresis, an x-tracta gel extractor (USA Scientific) was used to remove a band of SDS-PAGE gel at ~110–140 kDa. SDS-PAGE gel samples underwent trypsin digestion and TiO2 phosphopeptide enrichment. The peptides were than separated on a Waters nanoACQUITY (75 μm × 25 mm eluted at 300 nl/min) and then underwent mass spectrometry analysis on a LTQ Orbitrap mass spectrometer. The Mascot distiller (Matrix Science, Boston, MA) and Mascot search algorithm were used for database searching. The database searched was: SwissProt_2011_12. The confidence level was set to 95% within the Mascot search engine for protein hits based on randomness. Four independent experiments and samples underwent mass spectrometer analysis for identification of phosphorylation (tyrosine, serine, and threonine).

### Identification of tyrosine point mutations on Eph-B4 tyrosine phosphorylation

COS cells were transfected with 1 μg of pShuttle-IRES-hrGFP-2 expressing either empty vector, WT-EphB4-HA, or mutant-EphB4-HA. The mutant-EphB4-HA plasmids consisted of: Y574F-, Y581F-, Y590F-, Y596F-, Y614F-, Y653F-, Y730F-, Y736F-, Y774F-, Y806F-, Y821F-, Y837F-, Y906F-, or Y924F-substitution. After 24 hr starvation, the transfected Cos-7 cells were treated with Ephrin-B2/Fc (2 μg/ml) for 1 min. Cell lysates were then harvested and prepared. Whole cell lysates were then immunoprecipitated for the HA-tag. The IP sample underwent immunoblotting for both HA (Eph-B4) and pTyr using the primary antibodies anti-HA [Y-11] (Santa Cruz, #sc-805) and anti-pTyr [4G10] (Millipore, #05-321), respectively.

### Biotinylation of surface-expressed proteins

COS cells were transfected with 1 μg of pShuttle-IRES-hrGFP-2 plasmid containing either WT-Eph-B4-HA or Y774F-Eph-B4-HA constructs. EZ-Link Sulfo-NHS-SS-Biotin reagent (Thermo Scientific) was used according to manufacturer recommendations. Neutravidin agarose resin beads (Pierce) were used to pull down samples, centrifuged, and the whole cell lysate (WCL), flow through (FT), and surface fractions underwent Western blotting. The primary antibodies used were anti-HA (Roche, #11-867-423-001) and anti-HSP90 (Cell Signaling, #4874).

### Creation of Eph-B4-producing lentivirus

The pLenti-III-GFP-C vector (Applied Biological Materials) was utilized as the backbone for WT- and Y774F-Eph-B4 insertion. The WT-Eph-B4-HA and Y774F-Eph-B4-HA constructs were excised using *SpeI* and *EcoRV* (New England BioLabs) restriction enzymes after digestion with 10x NEBuffer 4 (New England BioLabs). Digested samples were then run on a 0.8% agarose gel and fragments corresponding to the pLenti-III-GFP-C (8.9 kb), WT-EphB4-HA (2.9 kb), and Y774F-Eph-B4-HA (2.9 kb) were excised and extracted using a gel extraction kit (Invitrogen).

The WT-Eph-B4-HA and Y774F-Eph-B4-HA purified products were then prepared for ligation by combining T4 DNA ligase (New England BioLabs) with 10x T4 DNA ligase buffer and the pLenti-IIIGFP-C vector and either the WT-Eph-B4-HA or Y774F-Eph-B4-HA inserts. The combined products were incubated overnight and transformed into *E. coli* (New England BioLabs). DNA was then extracted through a Miniprep spin kit (Qiagen). Samples underwent DNA sequencing to confirm WT- or Y774F-Eph-B4-HA insertion into pLenti-III-GFP-C.

### Production and purification of lentivirus

All steps were conducted in approved Biosafety Level-II areas using appropriate techniques and precautions. Early passage HEK 293 T cells (ATCC) were co-transfected with WT-Eph-B4-HA-pLenti-III or Y774F-HA-Eph-B4-HA-pLenti-III, an envelope vector, pMD2.g, and packaging vector, psPAX2 (Addgene). After co-transfection, the cells remained in a 37 °C, 5% CO_2_ incubator for 48 hr. The supernatant was then collected, centrifuged, and purified using a column-based Lenti-X Maxi Purification kit (Clontech). Titration of lentivirus was determined through the use of the Lenti-X qRT-PCR Titration kit (Clontech).

### Lentivirus treatment

An infrarenal aorto-caval AVF was created in WT mice as described above. After unclamping and confirming fistula flow, a 30% w/v f-127 pluronic gel (Sigma Aldrich) was used for lentiviral delivery of 2·10^6^ copies of either WT-Eph-B4 or Y774F-Eph-B4 to the AVF adventitial surface. After visual confirmation that the pluronic gel mixture had solidified, the abdomen was closed.

### Adenovirus treatment

Infrarenal aorto-caval AVF were created in WT mice as described above. After unclamping and confirming fistula flow, 1·10^6^ copies of commercially available adenovirus (Vector Biolabs, Malvern, PA) containing either constitutively active Akt1 (Ad-CMV-Akt1 (myr)), dominant negative Akt1 (Ad-CMV-Akt1 (dn)), or a control (Ad-CMV-Null) were applied to the AVF adventitial surface in a 30% w/v pluronic gel. After visual confirmation that the pluronic gel mixture had solidified, the abdomen was closed as described above.

### Statistics

All data were analyzed using Prism 7 software (GraphPad Software, Inc., La Jolla, CA). Comparison between groups was performed with two-tailed t-test or ANOVA with post hoc tests using the Sidak’s multiple comparison test. *P* values < 0.05 were considered significant.

## Electronic supplementary material

Supplementary Data File
