# Supplementary material for: Eph-B4 regulates adaptive venous remodeling to improve arteriovenous fistula patency
Source: Sci Rep. 2017 Nov 13;7:15386. doi: 10.1038/s41598-017-13071-2 (PMC5684317; doi:10.1038/s41598-017-13071-2)

SUPPLEMENTARY MATERIAL

Eph-B4 regulates adaptive venous remodeling to improve arteriovenous fistula patency

Clinton D. Protack MD, PhD,^†^ Trenton R. Foster MD,^†^ Takuya Hashimoto MD, PhD,^†^

Kota Yamamoto MD, PhD, Monica Y. Lee PhD, Jan R. Kraehling PhD,

Hualong Bai MD, Haidi Hu MD, PhD, Toshihiko Isaji MD, PhD, Jeans M. Santana,

Mo Wang MD, William C. Sessa PhD, and Alan Dardik MD, PhD

**Authorship note:** ^†^C.D. Protack, T.R. Foster, and T. Hashimoto contributed equally to this work.

Supplementary Figure 1

Supplementary Figure 1. Eph-B4 phosphorylation in mouse veins and rat patch neointima during adaptive remodeling. (A) Representative photomicrographs showing Eph-B4 (green), phosphotyrosine (pTyr; red), and merged (yellow) signal in AVF venous endothelium (rows 2 and 4) compared to sham (rows 1 and 3) in mouse aortocaval model. White arrows indicate unmerged Eph-B4 signal in AVF specimens. L = vessel lumen. (B) Bar graphs show quantification of immunoreactive signal (IF); *P* < 0.0001 (ANOVA). *, *P*< 0.05 (*P*<0.0001, AVF vs sham, day 7; post hoc); **, *P* < 0.05 (*P*=0.0426 AVF vs sham, day 21; post hoc). *n*=3-4. Scale bar, 100µm. (C) Representative photomicrographs (left panel) showing Eph-B4 (green), phosphotyrosine (pTyr; red), and merged (yellow) immunoreactive signal in endothelium (day 14) in rat patch model with AVF. White arrowheads indicate unmerged Eph-B4 signal. Bar graph (right panel) shows quantification of immunoreactive signal; *P* < 0.0001 (ANOVA). *, *P*< 0.05 (*P*<0.0001, Eph-B4 control vs AVF; *P*=0.0001, pTyr control vs AVF post hoc). *n*=3. Scale bar 100µm. Data represent mean ± SEM.

**Supplementary Figure 2**

**Supplementary Figure 2. Aortic remodeling in response to Eph-B4 stimulation. (A)** Line graph showing infrarenal aorta diameter in mice treated with control or Ephrin-B2/Fc; *P*=0.80 (ANOVA). *n*=4-5. Data represent mean ± SEM.

**Supplementary Figure 3**

**Supplementary Figure 3. Reduced Eph-B4 activity influences proliferation and aortic remodeling. (A)** Bar graph showing percentage of cells in the AVF venous limb positive for proliferation (Ki67), day 21; *, *P*=0.049 (t-test). *n*=4. **(B)** Bar graph showing percentage of cells in the AVF venous limb positive for or apoptosis (cleaved caspase-3), day 21. *P=*0.7133. *n=4.* **(C)** Line graph showing infrarenal aorta diameter in control or Eph-B4 het mice; *P*=0.38 (ANOVA). *n*=7-9. **(D)** Bar graph showing percentage of cells in the AVF venous limb wall positive for proliferation (Ki67), day 21. *P*=0.0098 (ANOVA). *, *P*<0.05 (*P*=0.0195, Y774F-Eph-B4 vs WT-Eph-B4; *P*=0.0153, Y774F vs control; post hoc). *n*=4. **(E)** Bar graph showing percentage of cells in the AVF venous limb wall positive for apoptosis (cleaved caspase-3), day 21. *P*=0.65 (ANOVA). *n*=4. **(F)** Line graph showing infrarenal aorta diameter in mice with AVF treated with WT-Eph-B4 (gray line) or mutant Y774F-Eph-B4 (blue line) compared to control (black line); **P*<0.0001 (ANOVA). *n*=5-11. Data represent mean ± SEM.

**Supplementary Figure 4**

**Supplementary Figure 4. Eph-B4 function is dependent on tyrosine 774 activity. (A)** Diagram showing locations of the Eph-B4 cytoplasmic tyrosines relative to the kinase domain. TM, transmembrane region; SAM, sterile alpha motif. **(B)** Results of mass spectroscopy analysis of COS cells treated with Ephrin-B2/Fc (3 min). **(C)** Representative Western blot showing loss of Eph-B4 phosphorylation in Eph-B4 receptors mutated in tyrosines 774, 821, or 924. HA, hyaluronidase; IP, immunoprecipitation; IB, immunoblot. n=3. **(D)** Western blot showing membrane localization of COS cells transfected with either wt-EphB4-HA-pShuttle or Y774F-EphB4-HA-pShuttle and then biotin labeled. Whole cell lysate (WCL), surface fraction (Surface), and flow through (FT). **(E)** Representative photomicrographs showing Eph-B4 (green), caveolin-1 (red), or merged (yellow) signal in COS cells treated with WT-Eph-B4 or mutant Y774F-Eph-B4 plasmids. White arrowheads show colocalization. **(F)** Representative Western blot (left panel) and bar graphs (right panel) showing Ephrin-B2/Fc stimulation of Akt and ERK 1/2 phosphorylation in COS cells transfected with WT-Eph-B4 or mutant Y774F-Eph-B4 plasmids. *, *P*=0.0016 (t test), pAkt (Ephrin-B2/Fc:control) WT vs Y774F. *n*=3. *, *P*=0.0252 (t-test), pERK1/2 (Ephrin-B2/Fc:control) WT vs Y774F. *n*=6. **(G)** Representative en face immunofluorescence showing Eph-B4 lentiviral expression on post-operative day 7 after pluronic gel delivery of 1x10^8^ copies of WT-Eph-B4-HA-pLenti-III. Intima (z1-3), media (z4). Data represent mean ± SEM.

**Supplementary Figure 5**

**Supplementary Figure 5. Akt1 activates eNOS during venous remodeling. (A)** Line graph showing infrarenal aorta diameter in WT mice treated with control adenovirus (control, black line), constitutively active Akt adenovirus (CA-Akt, red line), or dominant negative Akt adenovirus (DN-Akt, blue line); *P*=0.76 (ANOVA). *n*=4-5.  **(B)** Line graph showing infrarenal aorta diameter in WT (black) and Akt1 KO mice (blue). *P*=0.43 (ANOVA). *n*=8-14. **(C)** Line graph showing *eNOS* mRNA expression in venous limb of AVF; *P*<0.0001 (ANOVA). *, *P*<0.05 (*P*=0.0028 at day 7; post hoc). *n*=5-8. **(D)** Bar graph shows quantification of eNOS immunoreactive signal in AVF compared to sham; *P*=0.0001 (ANOVA). *, *P*< 0.05 (*P*=0.0082 at day 7; *P*=0.0071 at day 14; post hoc). *n*=8. **(E)** Bar graph shows quantification of eNOS immunoreactive signal in mice treated with control adenovirus, constitutively active Akt adenovirus (CA-Akt), or dominant negative Akt adenovirus (DN-Akt) at day 21; *P*=0.0036 (ANOVA). **P*=0.0038 control vs CA-Akt; ***P*=0.0114 CA-Akt vs DN-Akt; post hoc). *n*=3. Data represent mean ± SEM．

**Supplementary Figure 6**

Western blots shown in the manuscript are presented in uncropped full-size format of the membranes or LI-COR images.


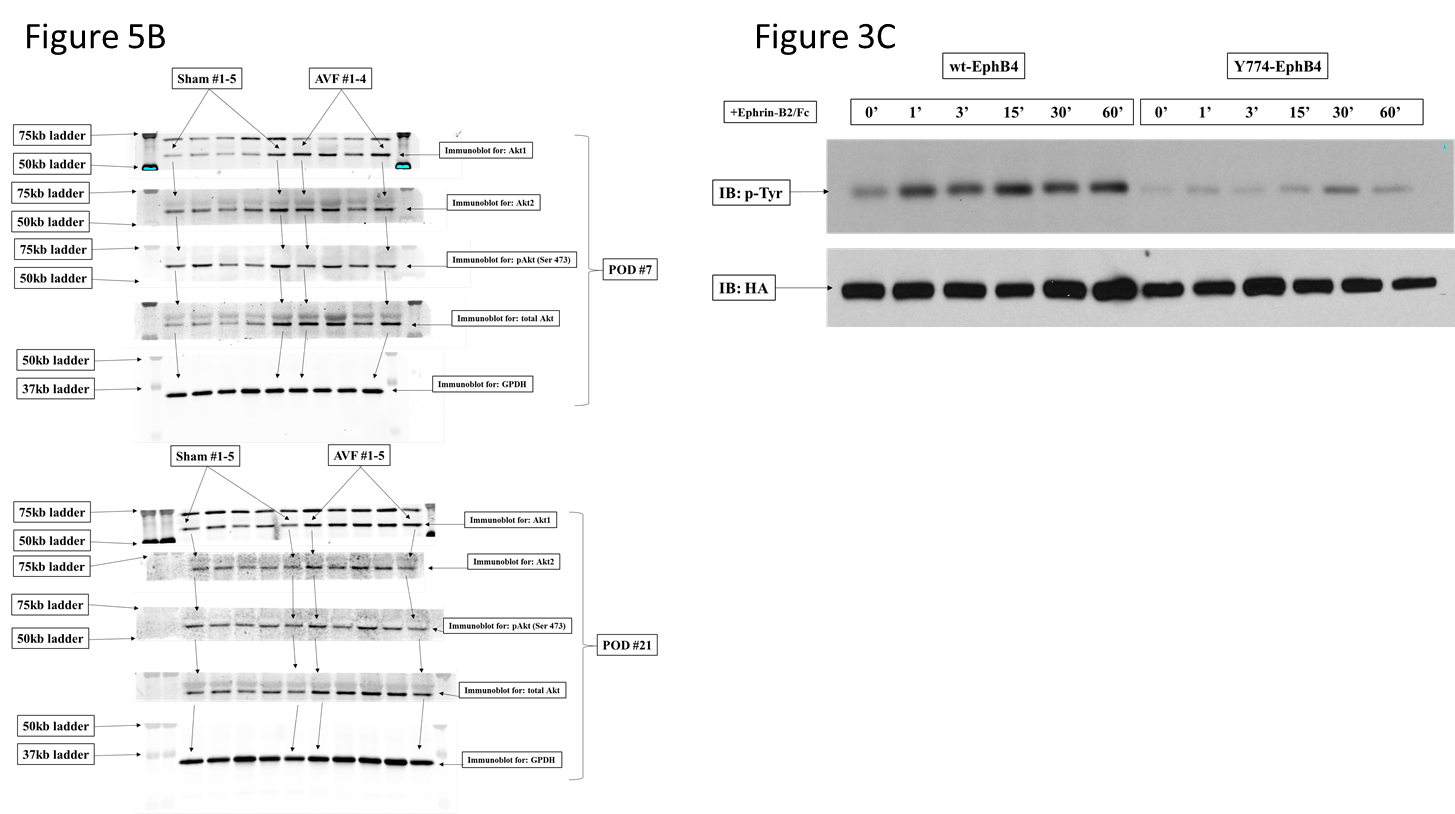

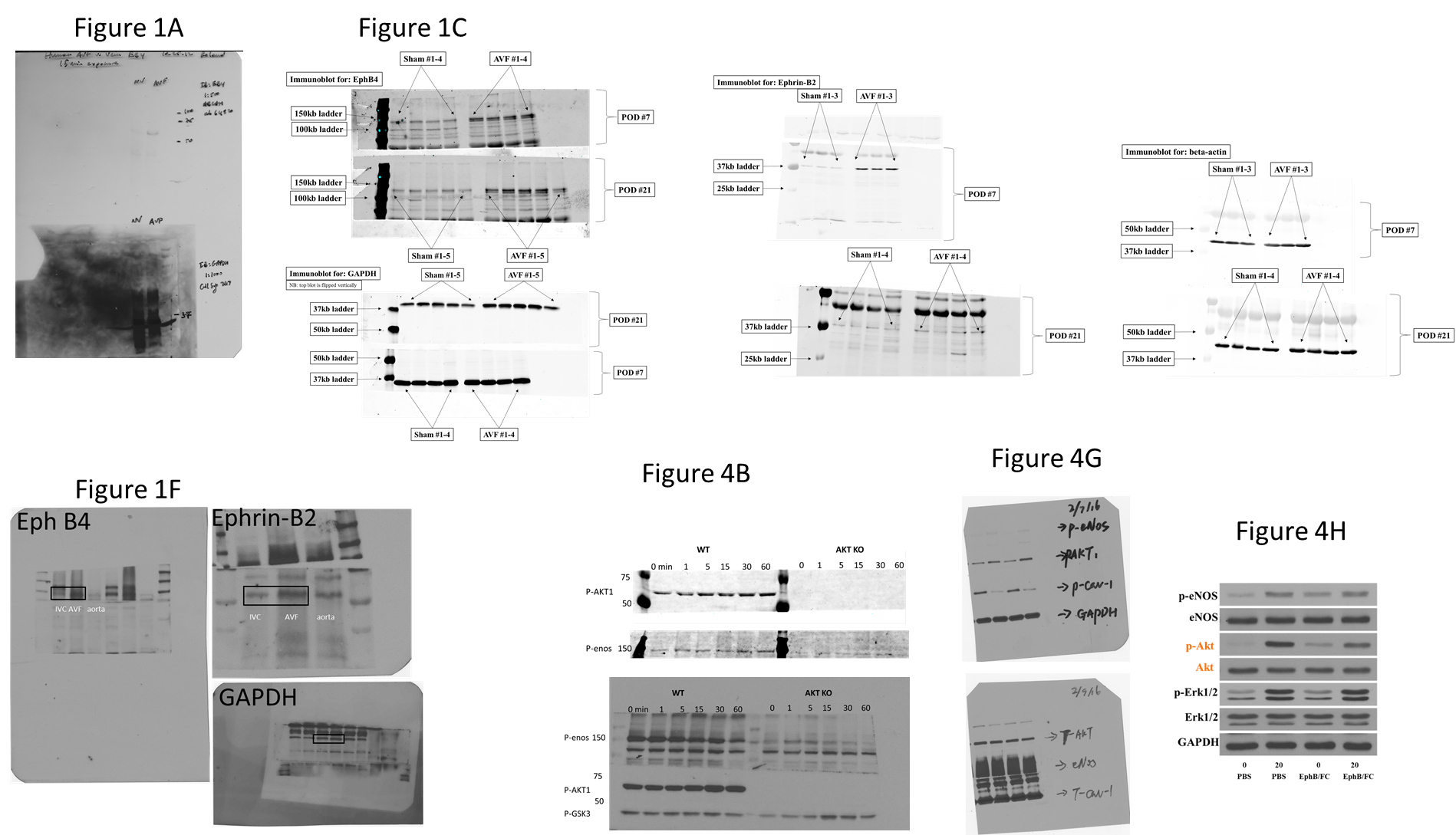

Supplement: Supplementary file 1 — Supplementary Data File [file 41598_2017_13071_MOESM1_ESM.doc]
